# Bacteriocin KvarM versus conventional antibiotics: comparative effectiveness in treating *Klebsiella pneumoniae* infections in murine intestinal models

**DOI:** 10.3389/fcimb.2025.1559865

**Published:** 2025-03-20

**Authors:** Indre Karaliute, Deimante Tilinde, Rima Ramonaite, Rokas Lukosevicius, Darja Nikitina, Jurga Bernatoniene, Irma Kuliaviene, Irena Valantiene, Dalius Petrauskas, Vilma Zigmantaite, Audrius Misiunas, Erna Denkovskiene, Ausra Razanskiene, Yuri Gleba, Juozas Kupcinskas, Jurgita Skieceviciene

**Affiliations:** ^1^ Institute for Digestive Research, Lithuanian University of Health Sciences, Kaunas, Lithuania; ^2^ Department of Drug Technology and Social Pharmacy, Lithuanian University of Health Sciences, Kaunas, Lithuania; ^3^ Department of Gastroenterology, Lithuanian University of Health Sciences, Kaunas, Lithuania; ^4^ Biological Research Center, Lithuanian University of Health Sciences, Kaunas, Lithuania; ^5^ Institute of Cardiology, Lithuanian University of Health Sciences, Kaunas, Lithuania; ^6^ Nomads UAB, Vilnius, Lithuania; ^7^ Nomad Bioscience GmbH, Biozentrum Halle, Halle (Saale), Germany

**Keywords:** *Klebsiella pneumoniae*, bacteriocin, klebicin, microbiome, murine models

## Abstract

**Introduction:**

The rapid emergence of multidrug-resistant bacterial species poses a critical threat by reducing the efficacy of antibiotics and complicating infection treatment. Bacteriocins, such as klebicin KvarM, have emerged as promising alternatives to traditional antibiotics due to their targeted antimicrobial activity. In this study, we evaluated the therapeutic potential of Eudragit-coated klebicin KvarM in a mouse model of *Klebsiella pneumoniae* intestinal colonization, assessing both its antimicrobial effectiveness and impact on commensal gut microbiota.

**Methods:**

Antimicrobial activity of KvarM in comparison to conventional antibiotic therapy with ciprofloxacin was tested in murine models for *K. pneumoniae* gastrointestinal (GI) tract infection. The haemolysin gene (*khe*) was chosen as the qualitative marker for *Klebsiella* genus identification, and 16S rRNA gene sequencing of V1-V2 hypervariable region was performed for analyses of gut microbiota.

**Results:**

Our results demonstrated that KvarM was highly effective in reducing *K. pneumoniae* colonization, showing the same efficacy as ciprofloxacin. Following *K. pneumoniae* inoculation, administration of KvarM resulted in a significant reduction in bacterial load indicating a 99% effectiveness. Furthermore, microbiome analysis of the gut microbiota revealed that KvarM therapy showed no significant changes in microbial composition compared with commensal microbiota composition, whereas administration of ciprofloxacin led to a significant decrease in microbial diversity.

**Discussion:**

These findings demonstrate that klebicin KvarM therapy is highly effective for treating intestinal *K. pneumoniae* infections and it does not affect the integrity of the gut microbiota.

## 1 Introduction

*Klebsiella pneumoniae* is a highly prevalent and antibiotic-resistant bacterium, known for causing frequent nosocomial outbreaks across Europe. As a member of the *Enterobacteriaceae* family, this Gram-negative opportunistic pathogen can colonize various parts of the human body, including skin, pharynx, and gastrointestinal tract. It primarily affects immunocompromised patients in hospitals, leading to bacteraemia, pneumonia, urinary tract infections or liver abscesses (Eger et al., 2021; Wang et al., 2020; Prokesch et al., 2016). According to the latest WHO reports, hypervirulent *Klebsiella pneumoniae* poses a significant threat even to healthy individuals, with resistance rates exceeding 50% in the European Region, placing it among the top-priority bacterial pathogens (World Health Organization, 2024a). Although treatment options for *K. pneumoniae* that is resistant to beta-lactamase or carbapenem are available, such as combining different antibiotics like aminoglycosides, imipenem, aztreonam, third generation cephalosporins, piperacillin/tazobactam or quinolones (Bassetti et al., 2018), the infection remains challenging to treat. Therefore, identification and validation of new treatment approaches is crucial in combating this pathogen.

Bacteriocins, narrow spectrum antimicrobial peptides produced by Gram-negative bacteria, have emerged as a potential alternative to conventional antibiotics (Simons et al., 2020). These peptides possess ability to selectively eliminate specific colonizing pathogen species or genus, and in theory, are expected to do so without disturbing the delicate balance of the host microbiota. While the largest group of antibiotics exert their function in a non-discriminative and broad way, bacteriocins are highly specialized peptides capable of recognition of species-specific receptors and pores on the outer membrane of Gram-negative bacterial cells allowing them to enter the periplasm or cytoplasm and kill the cell by creating pores in the inner membrane, inhibiting the cell wall synthesis or acting as DNAses/RNAses. This ability is usually limited to the members of the same species or genus (Denkovskienė et al., 2019).

The authors of the current study have previously discovered and characterized a group of colicin-like bacteriocins known as klebicins, specifically targeting pathogenic *Klebsiella* species (Denkovskienė et al., 2019). These small ribosomally synthesized peptides exert their action through pore formation and peptidoglycan degradation. Further, we have demonstrated the antibacterial efficacy of klebicins against *Klebsiella in vivo* using both non-mammalian and mammalian animal models (Karaliute et al., 2022). However, despite these significant findings, the further research of klebicins is still highly needed, due to lack of information on the most optimal formulation, as well as the effect of this therapy on the gut microbiome.

In this study, we aimed to evaluate antimicrobial activity of klebicin KvarM in a mouse model of intestinal colonization by *K. pneumoniae*. We tested dual release formulations using two different encapsulation strategies of KvarM for oral delivery: Eudragit^®^ S100 (dissolves in an alkaline environment and retain structural integrity in acidic conditions) to ensure the release in the large intestine, and Eudragit^®^ L100 (dissolves above pH 5.5 to prevent the degradation in acidic environments) - in the small intestine (Zhang et al., 2023). This approach enabled us to deliver klebicin KvarM to lower gastrointestinal tract and enhanced its potential to effectively eliminate *Klebsiella* bacteria. Furthermore, we compared the efficacy of KvarM to conventional antibiotics and investigated the impact of both antimicrobial compounds on the gut microbiota. To the best of our knowledge, this evaluation provides first experimental evidence that klebicins used in therapeutic doses provide little or no disturbances in the gut microbiome.

## 2 Materials and methods

### 2.1 Expression and purification of klebicin KvarM

For the production of KvarM, the klebicin – coding gene with plant-optimized codons was cloned into a magnICON^®^ tobacco mosaic virus-based vector pICH29912. The resulting binary expression vector was used to transform *Agrobacterium tumefaciens*. Subsequently, KvarM was transiently expressed in plants by infiltrating transformed agrobacteria strain into leaves of young *Nicotiana benthamiana* plants by vacuum. Following this, KvarM was purified to homogeneity from crude bacteriocin-containing plant extracts using a two-step protein chromatography process. The efficacy of bacteriocins were validated using different laboratory techniques and models. For more detailed information on the expression, purification and validation of bacteriocins please refer to our previous studies by I. Karaliute et al. (2022) and E. Denkovskienė et al. (2019).

### 2.2 Coating KvarM with Eudragit® L100 and Eudragit® S100

Due to the pH susceptibility and sensitivity of klebicin, KvarM was coated with Eudragit^®^ L100 and Eudragit^®^ S100 to achieve controlled pH release. The selection of this coating method was based on our previously published study assessing pH levels in the mouse gastrointestinal (GI) tract. Study revealed significant pH variations across different sections of the mouse GI tract (stomach: pH 3.3 ± 0.92; small intestine: pH 6.5 ± 0.34; large intestine: pH 7.4 ± 0.42). These measurements provided crucial information for choosing an appropriate Eudragit^®^ formulation (Karaliute et al., 2022). This coating strategy aims to protect the integrity of KvarM during transit through the GI tract. To obtain 5% solution of Eudragit^®^ L100, 100 mg of Eudragit^®^ L100 powder (Evonik Industries, Germany) was dissolved in 2 mL of 50 mM Phosphate buffer, and 2 M NaOH added until pH of the solution reached acidic levels (pH 6-7) for complete dissolution. To obtain 5% solution of Eudragit^®^ S100, 100 mg of Eudragit^®^ S100 powder (Evonik Industries, Germany) was dissolved in 2 mL of 50 mM Phosphate buffer and 2 M NaOH added until pH of the solution reached alkaline levels (pH 8), for complete dissolution.

Lyophilized KvarM samples (each of 0.25 mg) were dissolved in 250 µL of dH2O, then mixed with 250 µL of Eudragit^®^ L100 or Eudragit^®^ S100 5% solution. KvarM-Eudragit mixtures were acidified carefully by adding 2 M HCl up to pH 4-5, to obtain Eudragit polymerization, indicated by turning of clear solution to white suspension. The prepared samples were lyophilized and dissolved in dH2O before use.

### 2.3 Klebsiella pneumoniae cultivation

In this study, *K. pneumoniae* subsp. *pneumoniae* 43816^TM,^strain obtained from the Global Bioresource Center (ATCC), was used for all experiments. Bacterial cultures were grown and cultured according to the supplier’s recommendations in LB nutrient medium (Gibco by Life Technologies, USA) with ampicillin (final concentration 25 μg/mL). The ampicillin concentration was determined using the minimal inhibitory concentration test (**Supplementary Figure S1**).

### 2.4 Mouse models

**Figure 1:**
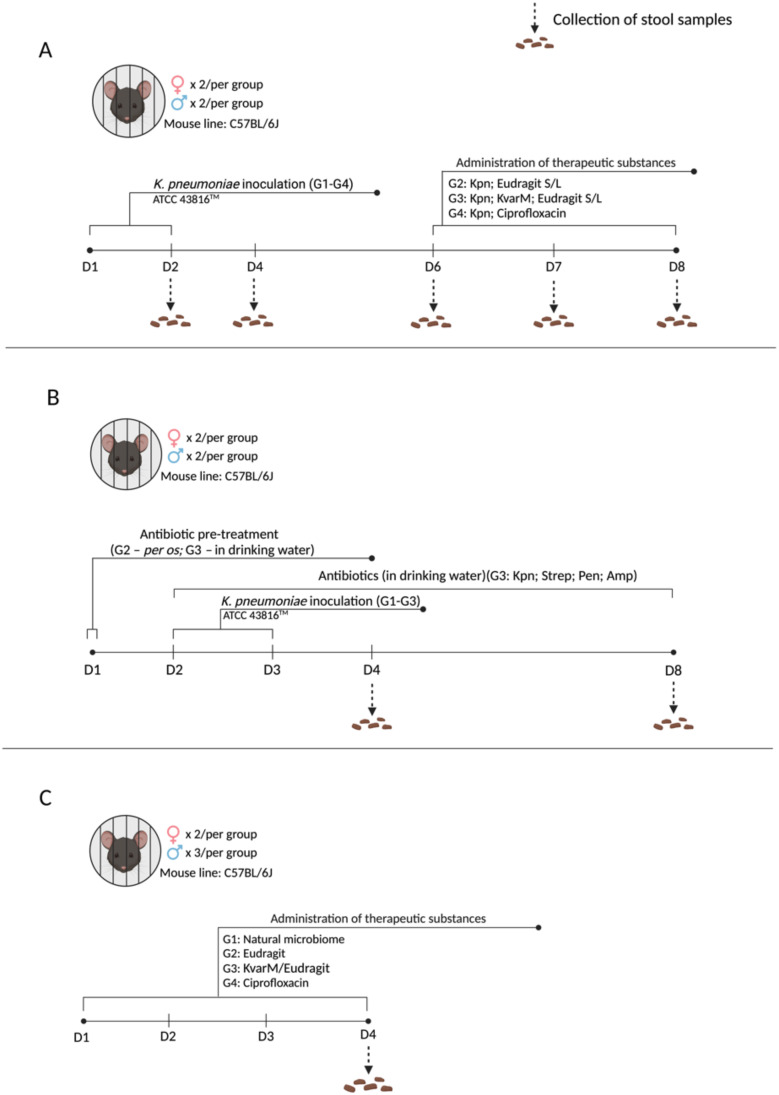
Experimental designs. **(A)** Effect of KvarM on *K pneumoniae*: All groups (G1-G4) were infected with *K pneumoniae* on days 1 and 2. On day 6 treatments were administered as follow: (Positive control; n = 4) did not receive any treatment; G2 (Kpn; Eudragit S/L; n = 4) received Eudragit^®^ S100 and Eudragit^®^ L100 treatment as a vehicle-only control; G3 (Kpn; KvarM; Eudragit S/L; n = 4) received Eudragit^®^ S100 and Eudragit^®^ L100 coated KvarM treatment; G4 (Kpn; Ciprofloxacin; n = 4) received ciprofloxacin therapy. **(B)** *K pneumoniae* infection model in antibiotic-pretreated microbiota: Group G1 (Positive control; n = 4) was infected with *K pneumoniae* on days 2 and 3. Groups G2 (Kpn; Strep; Pen; n = 4) and G3 (Kpn; Strep; Pen; Amp; n = 4) were given penicillin and streptomycin pre-treatment (day 1) before inoculation of *K pneumoniae* (days 2 and 3). Group G3 received low dose ampicillin therapy after bacterial inoculation (days 2-8). **(C)** Impact of KvarM on microbiome composition: Group G1 (Natural microbiota; n = 5) served as the control group without any interventions. Groups G2-G4 received daily treatments (days 1-4) as follows: G2 (Eudragit; n = 5) received Eudragit^®^ S100 and Eudragit^®^ L100 administration; G3 (KvarM/Eudragit; n = 5) received Eudragit-coated KvarM; G4 (ciprofloxacin; n = 5) received only ciprofloxacin therapy.

Three different animal study designs were used to perform a comprehensive evaluation of the effect of klebicin KvarM in experimental murine model of *K. pneumoniae* colonization (**Figure 1**). For these studies, 8–10 weeks old, C57BL/6J line, female (n = 22; 19–25 g) and male (n = 26; 22–27 g) mice were used (acquired from the Lithuanian University of Health Sciences vivarium of laboratory animals and Vilnius University vivarium of laboratory animals). All regulated procedures on living animals were approved by The Lithuanian Ethics Committee of Biomedical Research (Protocol no. G2-119).

To investigate the antimicrobial efficacy of Eudragit-coated KvarM, mouse model was established using *K. pneumoniae* inoculation (10^7^ CFU/50 μL) and administration of three different therapeutic substances (**Figure 1A**): (i) positive control group – inoculated with *K. pneumoniae* only (10^7^ CFU/50 μL); (ii) a vehicle control – Eudragit^®^ S100 (0.125 µg/μL) and Eudragit^®^ L100 (0.125 µg/μL) mix was inoculated to evaluate its impact on *K. pneumoniae*; (iii) therapy – Eudragit S100-coated KvarM (2.5 μg/μL) and Eudragit^®^ L100-coated KvarM mix (2.5 μg/μL) was used to examine its potential against *K. pneumoniae* gastrointestinal tract infection, and (iiii) antibiotic – ciprofloxacin (2 mg/200 μL) was used to compare its impact with KvarM efficacy against *K. pneumoniae* bacteria (**Supplementary Figure S3**). Two types of formulations of KvarM were used (Eudragit^®^ S100 and Eudragit^®^ L100), as *K. pneumoniae* bacteria usually colonize the upper and lower digestive tract. To prevent the dissolution of Eudragit coat in alkaline medium of mice oral cavity and release of antimicrobial substances before reaching upper and lower digestive tract, all the treatments were administered by gavage into the stomach. The safety of various klebicin concentrations was evaluated in a previous study by our group, guiding the selected dosages (Karaliute et al., 2022; Denkovskienė et al., 2019). To replicate natural bacterial colonization in mice, *K. pneumoniae* bacteria were orally inoculated using a pipette.

Finally, to evaluate the importance of microbiota during *K. pneumoniae* colonization, mouse model was created utilizing antibiotic treatment before and after bacteria inoculation (**Figure 1C**). Before introduction of bacteria, antibiotic streptomycin (targets Gram-negative bacteria; 2 mg/mL) and penicillin (targets Gram-positive bacteria; 2000 U/mL) were applied to induce an imbalance in the natural intestinal microbiota, facilitating the colonization of the mouse intestine by *K. pneumoniae* bacteria, followed by low-dose ampicillin therapy (targets Gram-positive and some Gram-negative bacteria; 125 mg/L) to maintain intestinal microbiome imbalance (**Figure 1B**). As well as the impact of ciprofloxacin (2 mg/200 μL) and KvarM/Eudragit^®^ S + L (500 μg/100 μL) on the murine microbiota was investigated (**Figure 1C**).

### 2.5 Nucleic acid extraction

Bacterial deoxyribonucleic acid (DNA) from rectum excrement samples were extracted using the AllPrep PowerFecal DNA/RNA Kit (Qiagen, Germany). Up to 200 mg of feces were used for the extraction procedures. The quantity and quality of extracted nucleic acids were evaluated by NanoDrop 2000 (Nanodrop Technologies, Wilmington, DE, USA) or Qubit 4 (Invitrogen, Carlsbad, CA, USA). All processes were completed upon the manufacturer’s instructions.

### 2.6 Quantitative assessment of Klebsiella pneumoniae using real time – PCR

The haemolysin gene (*khe*) was chosen as the qualitative marker for *Klebsiella* genus identification (Feng et al., 2023). The standard curve was created based on DNA samples of *K. pneumoniae* strain ATCC 43816™ to test the generated primers’ efficiency (**Supplementary Figure S2**). The quantitative real-time PCR (qRT-PCR) was performed using TaqMan Universal Master Mix II with UNG, TaqMan probe (5’-6-FAM-CGCGAACTGGAAGGGCCCG-TAMRA-3’), and primers (Forward: 5’-GATGAAACGACCTGATTGCATTC-3’, Reverse: 5’-CCGGGCTGTCGGGATAAG-3’ (Applied Bio systems, USA) following the manufacturer’s recommendations. The amplification of the *khe* gene was determined by ABI Fast 7500 System (Life Technologies, Carlsbad, CA, USA) according to the standard protocol. Positive control for DNA was isolated from *K. pneumoniae* and negative – isolated from *Escherichia coli*. For qRT-PCR 60 ng of DNA per reaction was used.

### 2.7 16S rRNA gene sequencing of V1-V2 hypervariable regions

In this study, *16S rRNA* gene sequencing was performed on an Illumina MiSeq device using the MiSeq V3 600-cycles reagent kit (Illumina, Inc., San Diego, JAV). Amplicons of the V1-V2 hypervariable region were generated using a custom library preparation protocol (for more detail see our previous publication (Gedgaudas et al., 2022)). Platinum SuperFi PCR Master Mix kit (Invitrogen, USA), nucleotide barcoded forward (27F) and reverse (338R) primers were used for library generation. Two technical replicates per sample were prepared. All the procedures were performed according to the manufacturer’s protocol and recommendations.

### 2.8 Bioinformatical and statistical analysis

Bioinformatic processing and statistical analysis were carried out in the R software environment as described previously (Camarinha-Silva et al., 2014). Briefly, the paired-end fastq files, devoid of barcodes and adapters, underwent quality checks, denoising, and preparation for subsequent analysis using the dada2 package [doi: 10.1038/nmeth.3869]. Annotation of bacterial sequences from the amplicon sequence variant (ASV) table was accomplished using the latest Silva database (version 138.1) (Quast et al., 2013). Data normalization and beta-diversity analysis at each taxonomical level were conducted with the DESeq2 package (Love et al., 2014). Significance was determined based on a corrected p-value (p.adj) < 0.05. Bioinformatic data processing and statistical analysis of the results were performed using the *RStudio* computational program.

*Friedman’s Test* and *Kruskal-Wallis test* were utilized to compare qRT-PCR data tendencies between the studied groups, afterwards using *Mann-Whitney U* test for non-parametric data points. Results were considered statistically significant when p ≤ 0.05.

## 3 Results

### 3.1 Klebicin KvarM reduces colonization of Klebsiella pneumoniae in the gut

**Figure 2:**
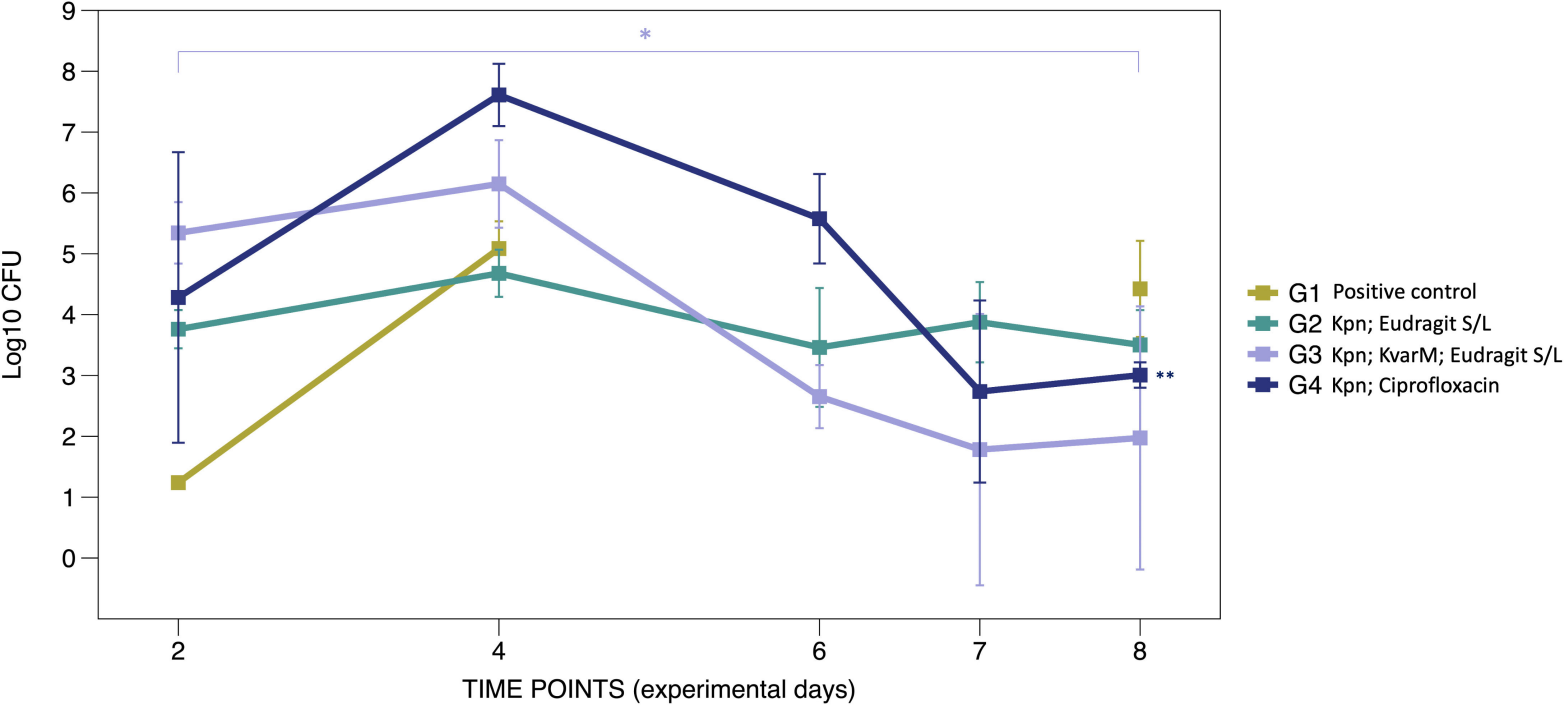
KvarM and antibiotic effectiveness (Study A). Using qRT-PCR the *K. pneumoniae* specific gene *khe* was amplified demonstrating the changes before and after KvarM and ciprofloxacin therapies, day 2 marks the first sample collection point after the initial bacterial inoculation procedures. Group G1 (positive control group) had been administered with *K. pneumoniae* (av. 2.17 x 10^8^ CFU on day 4) without subsequent therapies, and the bacterial count was evaluated on the day 8 (2.60 x 10^9^ CFU). Vehicle control group G2 (Kpn; Eudragit S/L) were inoculated with *K. pneumoniae* (day 4 av. 1.83 x 10^7^ CFU) and followed by administration of Eudragit^®^ S/L (day 8 av. 1.00 x 10^7^ CFU). KvarM therapy group G3 (Kpn; KvarM; Eudragit S/L) showed an average of 1.4 x 10^6^ CFU after *K. pneumoniae* inoculation (day 4) and average of 93.0 CFU after KvarM therapy on day 8 (*p = 0.003). Antibiotic therapy group G4 (Kpn; Ciprofloxacin) showed an average of 6.03 x 10^7^ CFU after bacterial inoculation on day 4 and average of 1.25 x 10^2^ CFU (**p = 0.0419) after ciprofloxacin therapy on day 8. The effectiveness was evaluated by comparing samples from the same group at different time points, considering the varying levels of bacterial colonization among the groups.

To assess the efficacy of KvarM therapy in *K. pneumoniae* infection, C57BL/6J mice were orally inoculated with *K. pneumoniae* subsp. *pneumoniae* strain (ATCC 43816™) for three consecutive days, followed by three different therapeutic interventions (**Figure 1A**). Administration of the KvarM treatment subsequent to *K. pneumoniae* colonization resulted in a 99.9% effectiveness, with an average of 1.4 x 10^6^ CFU after inoculation and 94 CFU after completion of KvarM therapy on the last day of the experiment (p = 0.041), demonstrating a consistent trend throughout the duration of the therapy. Furthermore, a comparison of effectiveness of KvarM and antibiotic ciprofloxacin therapies revealed that KvarM therapy exhibits similar effectiveness as antibiotics, achieving 99.9% reduction in bacterial load (4.08 x 10^7^ CFU after bacterial inoculation and 1.01 x 10^3^ CFU after therapy) (**Figure 2**). Moreover, the effectiveness of ciprofloxacin remained consistent throughout the study period.

### 3.2 The impact of antibiotics on mice microbiota during Klebsiella pneumoniae colonization

To elucidate the role of the microbiota during bacterial colonization and infection, we evaluated the impact of antibiotic treatment abundance of *K. pneumoniae* in the feces. In order to evaluate the impact of antibiotics, we conducted an experiment using *K. pneumoniae* inoculation and utilizing antibiotic pretreatment (G2) followed by low-dose antibiotic therapy (G3) (**Figure 1B**).

**Figure 3:**
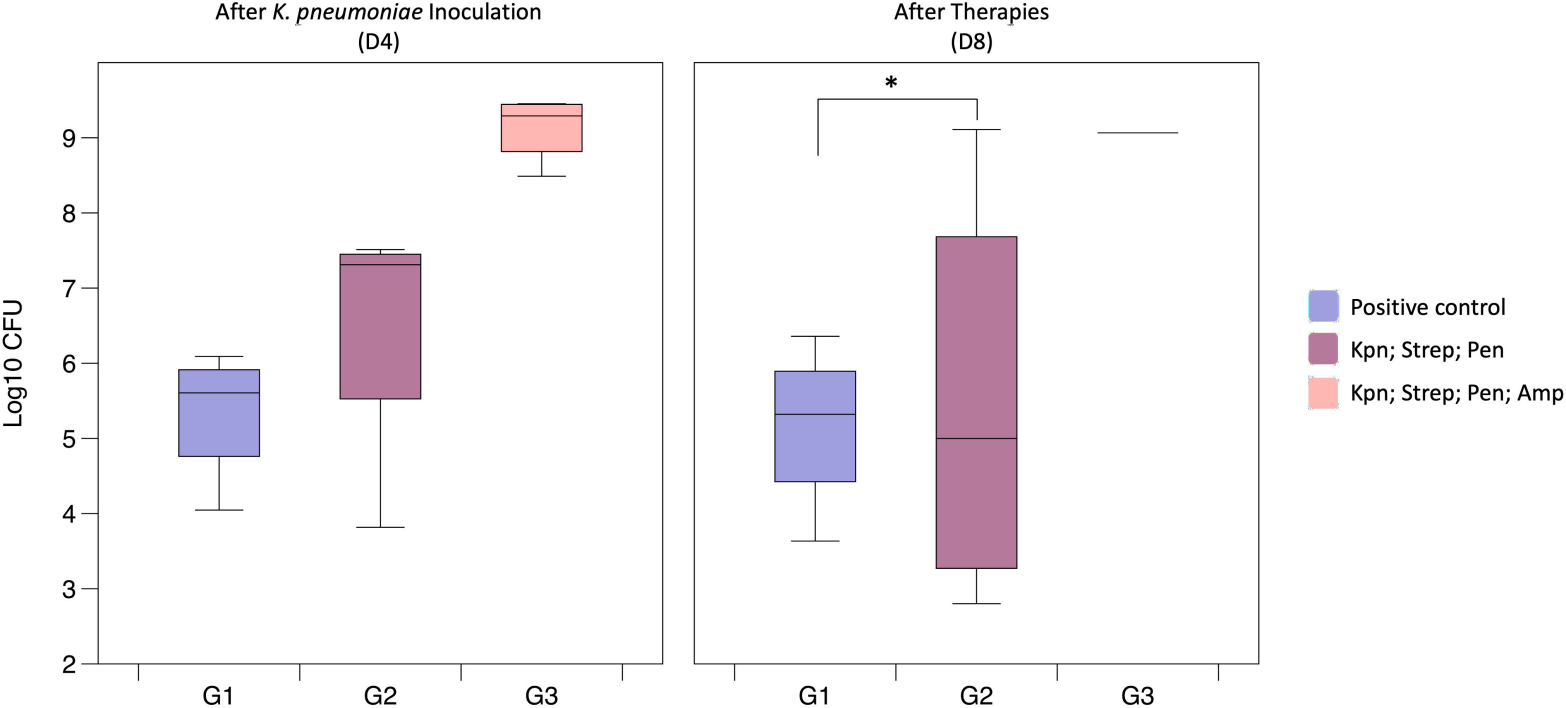
Gut colonization of *K. pneumoniae* following antibiotic treatment (Study B). The G1 (Positive control) had been administered with *K. pneumoniae* (9.72 x 10^8^); Group G2 (Kpn; Strep; Pen) were pre-treated with streptomycin and inoculated with *K. pneumoniae* (after pre-treatment av. 1.58 x 10^8^ CFU); Group G3 (Kpn; Strep; Pen; Amp) showed an av. of 10.7 x 10^7^ CFU after bacterial inoculation and av. of 9.41 x 10^8^ CFU after therapies (*p = 0.001).

**Figure 4:**
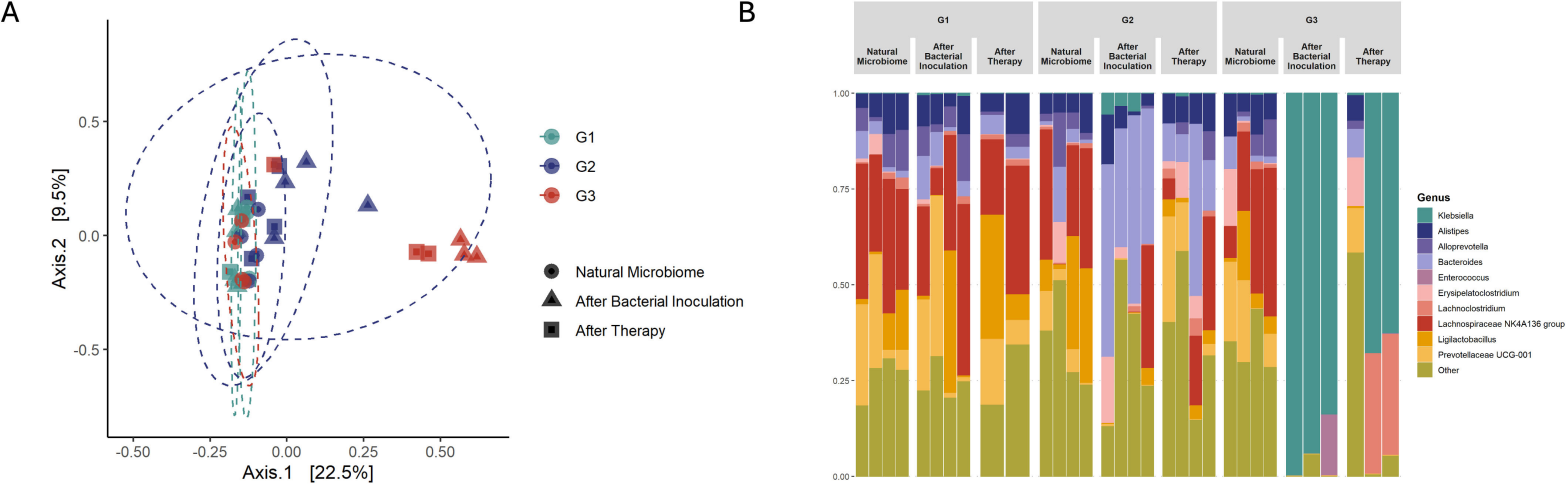
Microbiota changes after antibiotic treatment (Study B). **(A)** in PCoA, red colored points are closer together representing group G3 (Kpn; Strep; Pen; Amp), which was pre-treated with antibiotics and after bacterial colonization receive low dose ampicillin therapy. As well as green and blue colored dots cluster together representing G1 (Positive control) and antibiotic pre-treated group G2 (Kpn; Strep; Pen). **(B)** Microbiota composition analysis showed the main changes in group G3 (Kpn; Strep; Pen; Amp) which was given antibiotic pre-treatment and small dosage of antibiotic therapy after bacterial inoculation (Day 4). In the figure Natural Microbiome refers to Day 1, before any procedures were carried out, and After Therapy refers to the last day of the experiment, Day 8.

After antibiotic pre-treatment, colonization of *K. pneumoniae* bacterial counts were slightly higher (3.09 x 10^6^ CFU) compared to the positive control group (2.18 x 10^5^ CFU), however the difference was not statistically significant (p = 0.343) (**Figure 3**). However, administering of antibiotics before, during, and after bacterial inoculation resulted in even significantly higher detectable *K. pneumoniae* colonization (9.41 x 10^7^ CFU, p = 0.029) with a clear trend seen in the **Figure 3**. Principal Coordinates Analysis (PCoA, UniFrac unweighted) supported the notion that microbiota imbalances after treatment with antibiotics enhances *K. pneumoniae* colonization in the gastrointestinal tract (grouping of mice in G3 before and after antibiotic treatment (**Figure 4A**)). Subsequent compositional analysis of the three groups revealed that the gut microbiome was highly colonized (1.35 x 10^9^ CFU, p = 0.002) with *K. pneumoniae* after antibiotic pre-treatment followed by low-dose antibiotic therapy (G3), with *Klebsiella* (p = 4.67 x 10^-49^) being the dominant species, followed by *Lachnoclostridium* (p = 1.74 x 10^-42^) (**Figure 4B**).

### 3.3 Klebicin KvarM therapy does not affect composition of microbiota in mice compared to antibiotic treatments

Finally, we evaluated the compositional changes in mice microbiome after bacteriocin KvarM and antibiotic ciprofloxacin therapies (**Figure 1C**). We observed distinct differences in microbial communities in mice subjected to antibiotic treatment and bacteriocin therapy. Following antibiotic administration, there was a significant change in microbial composition, with a notable decrease in the abundance of several taxa. Specifically, Alistipes (p = 6.87 x 10^-28^), a constituent of the commensal microbiome, exhibited a pronounced decline, indicating impact of a broad-spectrum antibiotic on the microbiota.

**Figure 5:**
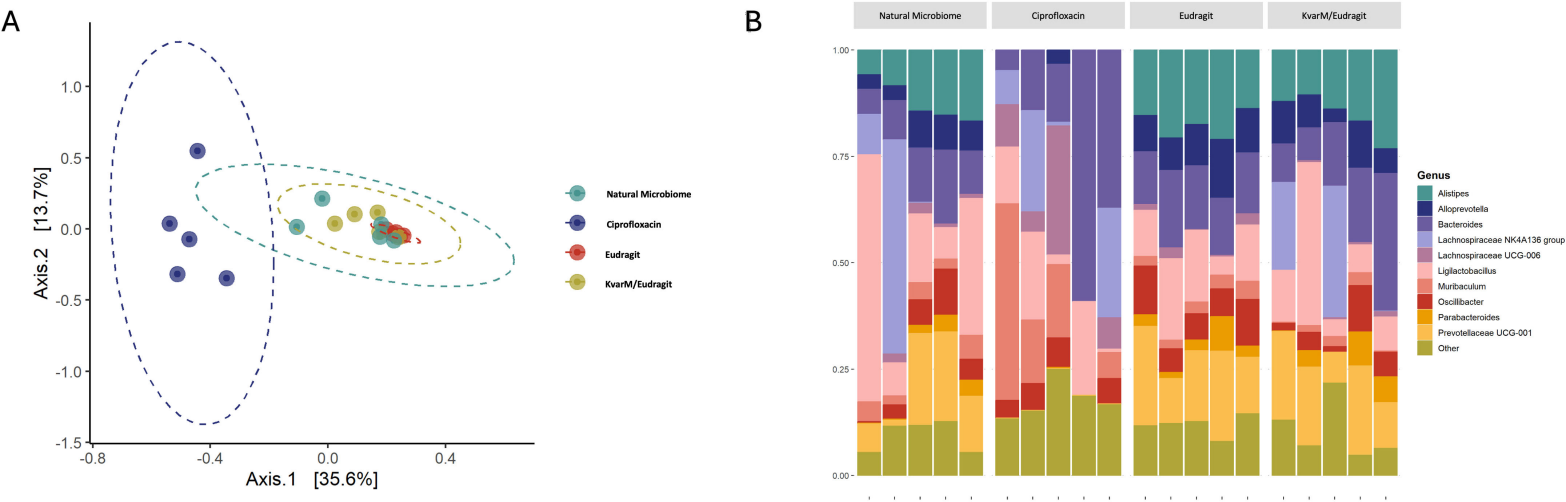
Microbiota changes during different therapies (Study C). **(A)** in PCoA, blue colored points are closer together representing antibiotic treated group G4 (Ciprofloxacin). Other groups (G1-G3) clustered together showing no significant changes in microbiome. **(B)** Microbiota composition analysis showed the main changes in antibiotic treated group G4 (Ciprofloxacin), all other groups showed no significant differences in bacterial composition.

Conversely, in mice treated with bacteriocin therapy, we observed minimal changes in the fecal microbial composition. This was complemented by observed significant changes between natural microbiome and antibiotics (p = 0.01), and between KvarM and antibiotics (p = 0.009) (**Supplementary Table S2**), based on the PCoA diversity plot (**Figures 5A, B**). Notably, key taxa (Prevotellaceae, Rikenellaceae, Tannerellaceae, Desulfovibrionaceae, Deferribacteraceae, Eggerthellaceae, Ruminococcaceae, Sutterellaceae, Clostridiaceae, Erysipelotrichaceae) involved in maintaining gut homeostasis appeared largely unaffected by bacteriocin treatment, highlighting the specificity and targeted nature of this therapeutic approach, whereas the abundance of these taxa was decreased in the group of antibiotics usage. These findings point out the potential of bacteriocin therapy as a modality for preserving the integrity of the fecal microbiome during the treatment of *K. pneumoniae* infection.

## 4 Discussion

The escalating global concern regarding antibiotic-resistant strains of *K. pneumoniae* necessitates innovative strategies to combat colonization and infection (World Health Organization, 2024b; Bassetti et al., 2018). In this study, we investigated the efficacy of the bacteriocin KvarM in treating *K. pneumoniae*-induced intestinal infection, utilizing an experimental C57BL/6J mouse line and commercial *K. pneumoniae* strain (ATCC 43816™) as a model system.

Our study explores bacteriocins, narrow-spectrum antimicrobial peptides produced by bacteria, as a potential alternative to traditional antibiotics. In previous studies, our group has already demonstrated that narrow-spectrum, colicin-like bacteriocins selectively target closely related bacterial species and are efficient in prolonged reduction of bacterial colonization over time, suggesting its potential for longer-lasting therapeutic effects (Karaliute et al., 2022). In this study, our primary objective was to establish a model for evaluating bacterion’s KvarM efficacy. We chose KvarM, as in previous study by one of the current study co-author’s E. Denkovskiene this bacteriocin has been shown to have a narrow spectrum of action *in vitro* and to be effective only against different strains of the *Klebsiella* genus (Denkovskienė et al., 2019). Comprehensive analysis with different isolates of *Klebsiella* genus bacteria (*K. pneumoniae*, *K. quasipneumoniae*, *K. oxytoca*, *K. variicola*, and *K. aerogenes*) indicated that KvarM reduced the number of bacteria from 1000 to 10000 times in liquid environment, and more than 1000 times in biofilm testing. These results directly correlate with the findings observed in our mouse models of *K. pneumoniae* intestinal colonization. We compared klebicin KvarM to ciprofloxacin in treating gastrointestinal infections. Ciprofloxacin significantly decreased bacterial loads (broad spectrum activity), some persistence was observed. This is likely due to antibiotic tolerance, incomplete eradication, or microbiome-mediated survival mechanisms (Anderl et al., 2000). As expected, KvarM effectively and consistently throughout the duration of the therapy reduced bacterial counts and thereby, resulted in a 99.9% effectiveness. The demostrated efficacy of KvarM was comparable to ciprofloxacin. The high effectiveness narrow-spectrum proteins has been shown in other bacterial colonization models as well. A study by N. Carpena used a murine model of *E. coli* colonization. By employing encapsulation strategies, a localized and sustained release of the protein antibiotics was achieved, resulting in a notable reduction in *E. coli* colonization (Carpena et al., 2021). These findings collectively contribute to the advancement of tailored antimicrobial therapies with enhanced efficacy and minimized side effects.

Understanding the colonization dynamics of *K. pneumoniae* was crucial for our investigation. The microbiome sequencing data analysis disclosed that pre-introduction of antibiotic therapy (streptomycin/penicillin) resulted in more efficient bacterial colonization compared to groups (Study B group G2) where antibiotic therapy was not applied before bacterial introduction. These findings align with other studies establishing *K. pneumoniae* infection models. In a study by T. M. Young it was noted that the *K. pneumoniae* isolate MKP103 more efficiently colonized mouse intestines when antibiotic therapy was applied before *K. pneumoniae* introduction. Antibiotic administration disrupted the mouse intestinal microflora, leading to prolonged *K. pneumoniae* infection for over 15 days (Young et al., 2020). Finally, we evaluated the changes of the fecal microbiome in response to antibiotic treatment and bacteriocin KvarM therapy. We observed no alterations in the composition of the microbiome using KvarM therapy, in contrast with the significant reduction in diversity following antibiotic administration, these findings highlight the specificity and targeted nature of bacteriocins. The results indicate that KvarM did not negatively affect the mouse intestinal microbiota, whereas ciprofloxacin significantly reduced microbiota’s alpha diversity. A. Palleja reported that compositional changes in the microbiota take up to six months to restore gut microbial alpha diversity to its original levels after exposure to antibiotics (Palleja et al., 2018), whereas our study showed that KvarM minimizes the impact on the conventional microbiome.

In conclusion, these findings indicate that klebicin KvarM therapy is highly effective in treating intestinal *K. pneumoniae* infections while having minimal impact on the gut microbiota composition.

## Data Availability

The datasets presented in this study can be found in online repositories. The names of the repository/repositories and accession number(s) can be found in the article/**Supplementary Material**.
