# Supplementary material for: Bacteriocin KvarM versus conventional antibiotics: comparative effectiveness in treating Klebsiella pneumoniae infections in murine intestinal models
Source: Front Cell Infect Microbiol. 2025 Mar 20;15:1559865. doi: 10.3389/fcimb.2025.1559865 (PMC11965673; doi:10.3389/fcimb.2025.1559865)
Supplement: Supplementary file 1 [file DataSheet1.pdf]

Supplementary Material

Comparative Efficacy of Bacteriocin KvarM against *Klebsiella pneumoniae* in a Murine Intestinal Model

Indre Karaliute, Deimante Tilinde, Rima Ramonaite, Rokas Lukosevicius, Darja Nikitina, Jurga Bernatoniene, Irma Kuliaviene, Irena Valantiene, Dalius Petrauskas, Vilma Zigmantaite, Audrius Misiunas, Erna Denkovskiene, Ausra Razanskiene, Yuri Gleba, Juozas Kupcinskas, Jurgita Skieceviciene.

1. METHODS

***Klebsiella pneumoniae* cultivation**

Minimal inhibitory concentration test used for the determination of ampicillin concentration.

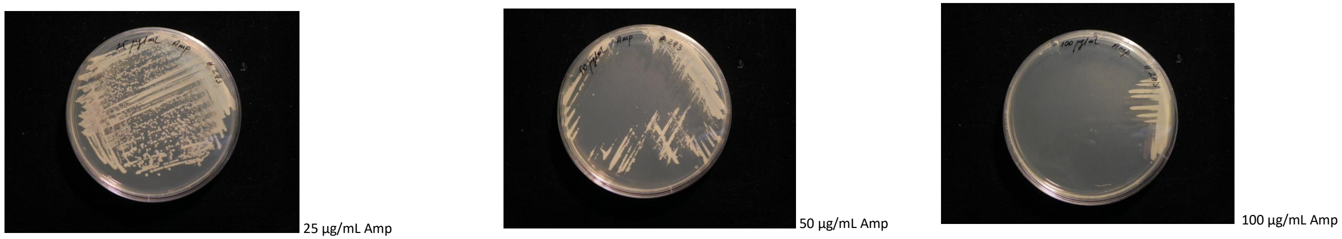

Supplementary Figure 1 *K. pneumoniae* resistance to ampicilin. *K. pneumoniae* strain ATCC 43816 resistances to ampicilin from 25 ug/mL to 100 ug/mL.

DNA-based standard curve for *K. pneumoniae* detection. 10^2, 10^3, 10^5, 10^6, 10^8, 10^9 and 10^{10} CFU of *K. pneumoniae* in 200 µl were subjected to DNA extraction with QIAamp Fast DNA Stool Mini Kit (protocol for liquid sample). DNA was eluted in 50 µl and 2 µl of DNA of each sample were taken for qRT-PCR. qRT-PCR procedures were performed as described in the methods section (see the manuscript).

DNA-based standard curve. qRT-PCR was validated by setting up the standard curves for detection of *K. pneumoniae* strains ATCC 12657 and ATCC 43816 using *khe* gene. As demonstrated in Fig. 1, the standard curves showed the expected linear relationship between the CT values and the concentration of bacteria cells at the DNA level.

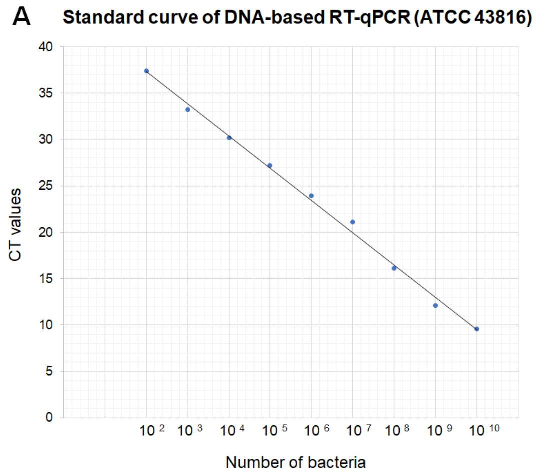

Supplementary Figure 2 Standard curves for the detection of *K. pneumoniae*. A – DNA-based standard curve of *K. pneumoniae* strain ATCC 43816 ($y = -3.4797x + 44.31$ with R^2=0,9959). The standard curves were generated by plotting the mean CT values in the range of bacteria concentrations from 10^2 CFU to 10^{10} CFU.

ANTIBIOTIC SENSITIVITY

The correct antibiotic for the experimental designs was chosen depending on *K. pneumoniae* strain ATCC 43816 resistances.

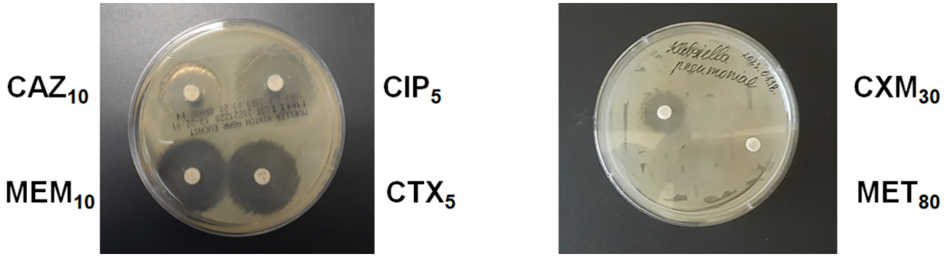

Supplementary Figure 3 Antibiotic sensitivity test. MEM_{10} (meropenem) inhibitory zone diameter – 30 mm; CIP_5 (ciprofloxacin) inhibitory zone diameter - 30 mm ; CAZ_{10} (ceftazidime) inhibitory zone diameter - 25 mm; CTX_5 (cefotaxime) inhibitory zone diameter – 29 mm ; CXM 30 (cefuroxime) inhibitory zone diameter – 19 mm; MET 80 (metronidazole) inhibitory zone diameter – 7.5 mm

Supplementary 1 table Antibiotic sensitivity test

Antibiotics	Inhibitory zone diameter (mm)
MEM_{10} (meropenem)	30
CIP_5 (ciprofloxacin)	30
CAZ_{10} (ceftazidime)	25
CTX_5 (cefotaxime)	29
CXM 30 (cefuroxime)	19
MET 80 (metronidazole)	7.5

2. RESULTS

Klebicin KvarM therapy does not affect composition of microbiota in mice compared to antibiotic treatments

The biostatistical analysis using Permanova test was completed to see the differences in microbiome between groups.

Supplementary 2 table Values of the Permanova analysis using biostatistical tool R

	Phylum		Class		Order		Family		Genus		Species		ASV	
	p value	F	p value	F	p value	F	p value	F	p value	F	p value	F	p value	F
**Natural microbiome group vs Ciprofloxacin group**	0,729	0,1757	0,867	0,1583	0,706	0,3416	0,171	1,6907	0,013	3,4559	0,006	10,145	0,01	3,7178
**KvarM+Eudragit groups vs Cirpofloxacin group**	0,178	2,3788	0,42	0,9331	0,312	1,345	0,04	2,7499	0,012	4,1582	0,007	8,0709	0,009	4,5703

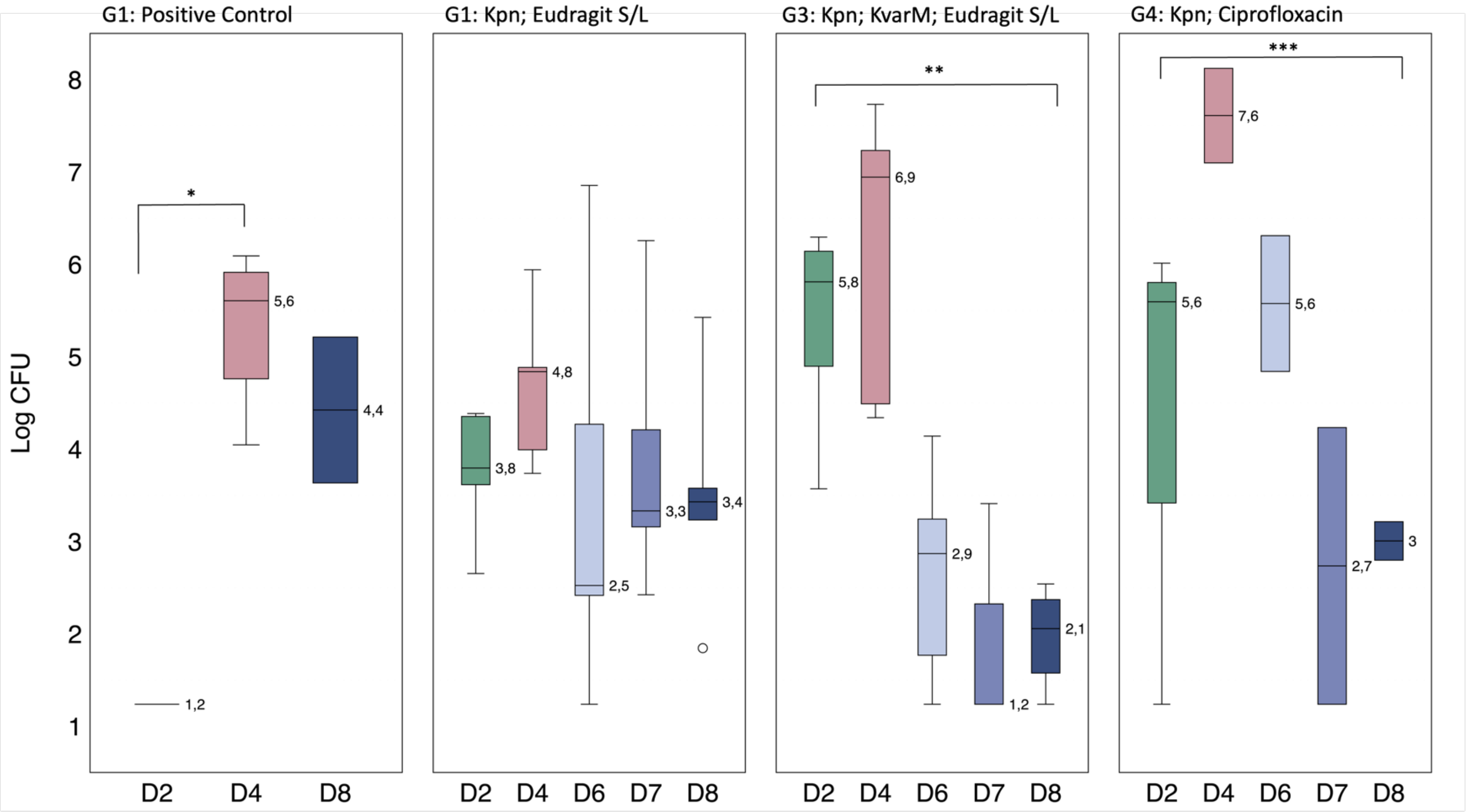

Supplementary Figure 4 Changes of bacterial counts within each group (Study A). Box plot representation of statistical differences across time points for G1 (Positive Control), G2 (Kpn; Eudragit S/L), G3 (Kpn; KvarM; Eudragit S/L), and G4 (Kpn; Ciprofloxacin). **G1 shows a significant increase from D2 to D4 (*p = 0,0028)**, indicating a strong early response. **G2 displayed no statistically significant differences between time points,** indicating a stable response. **G3 exhibits multiple significant fluctuations**, with highly significant differences between **D2 and D7/D8 (**p < 0,0035)**, suggesting a dynamic progression over time. **G4 shows a late-phase decline between D4 and D8 (***p = 0,0419)**.
